# Factors Affecting Macro-Structural Development in the Cerebral Cortex: The Potential Role of Tissue Removal Through Pruning and Apoptosis

**DOI:** 10.3390/biology14121651

**Published:** 2025-11-23

**Authors:** Jacob Levman

**Affiliations:** 1 Department of Computer Science, St. Francis Xavier University, Antigonish, NS B2G 2W5, Canada; jlevman@stfx.ca; 2 Nova Scotia Health Authority, Halifax, NS B3H 1V8, Canada; jacob.levman@nshealth.ca

**Keywords:** cortex, folding, neurodevelopment, pruning, thinning, sulcal development, apoptosis

## Abstract

Several theories have been developed to explain mechanisms of macro-structural development in the cerebral cortex, including external skull constraints, axonal tension, differential proliferation of neural progenitors, differential cortical expansion, and axonal pushing. These theories are not necessarily mutually exclusive, and some combination thereof may be required in order to fully explain and characterize complex folding, sulcal development and thinning in the cortex. This manuscript provides an overview of the leading theories of contributing factors to macro-structural cortical development, and presents additional potential contributing factors, including tissue removal through pruning and apoptosis. Although tissue removal has been proposed as a potentially major factor in microcephaly and megalencephaly—conditions with major deviations from healthy macro-structural cortical development—in this manuscript, it is proposed that tissue removal may be an important factor in healthy neurodevelopment, as well as in additional pathological conditions. This manuscript also presents the theory that tissue removal may be linked to learning. Potential consequences for a variety of pathological conditions, and potential relationships with previously established theories of macro-structural cortical development are discussed.

## Simple Summary

Several theories have been developed to explain major aspects of brain development, including regional differences in growth rates, the constraining effects of the skull, and tension and pushing within neural tissue. This paper reviews these theories and develops the hypothesis that tissue removal also potentially plays an important role in brain development. Tissue removal’s potential relationship with learning is also presented. Potential implications for a variety of developmental disorders are also discussed.

## 1. Background

Several theories and experiments have been proposed to explain macro-structural cortical development (MSCD), inclusive of sulcal formation and complex folding as well as gyrification and cortical thinning. These include mechanical folding by regional differential growth [1,2,3], skull constraints [4], axonal tension [5,6], differential proliferation of neural progenitors [7], differential cortical expansion related to cytoarchitecture [8,9], and axonal pushing [10]. For a thorough review of early cortical folding and sulcal formation, please see [11]. These theories are not necessarily mutually exclusive, and some combination thereof is expected to contribute to the complex folding and development observed in the human cerebral cortex. The reader is directed to reviews on cortical gyrification [8,12]. It should be noted that major sulcal formation and complex folding occur primarily during gestation [13], whereas gyrification and cortical thickness changes have been reported throughout the human lifespan [14,15,16,17], which includes my own work, inclusive of cortical thickness analyses in a pediatric population [16].

It should be noted that a study on the pathological conditions microcephaly (characterized by unusually small brains) and megalencephaly (characterized by unusually large brains) proposed that apoptosis, or programmed cell death, may be dysregulated, potentially contributing to the abnormal macro-structural presentation of the brain in these conditions [18]. Indeed, mutation of key genes linked with apoptosis has been implicated in microcephaly [19] and megalencephaly [20]. Thus, abnormalities of tissue removal through apoptosis have been potentially implicated in extreme examples of neurodevelopmental disorders as manifested at the macro-structural level. In this paper, the theory that tissue removal (through apoptosis and pruning) plays a potentially important role in both healthy MSCD and MSCD in a variety of pathological conditions is presented. The potential role of tissue removal in learning is also considered.

Apoptosis and pruning are the primary mechanisms considered in this analysis for neurological tissue removal and its potential effect on MSCD. Programmed cellular death is normally a healthy natural procedure. In the brain, neuronal apoptosis has been associated with pruning [21,22,23,24], which may reflect the removal of connections to and from removed apoptotic neurons. Pruning includes a diverse collection of subtypes, including synaptic, dendritic, axonal, and microglial pruning. Synaptic pruning involves the removal of synapse tissue, which can occur in the absence of apoptosis. Considerable evidence suggests synaptic pruning is activity-dependent [25], occurs throughout the lifespan, and is potentially implicated in neurodevelopmental diseases [26]. Microglia are immune cells found in the central nervous system that “prune developing synapses and regulate synaptic plasticity and function” [27]; thus, microglia represent a mechanism for synaptic pruning. Dendritic and axonal pruning “facilitate the removal of exuberant neuronal connections” [28], and “axon pruning involves the removal of axons that had already made synaptic connections; thus, axon pruning is tightly associated with synapse elimination” [28]. Average axon density has been reported to decrease across ages [29], implying that axonal and dendritic pruning occur across the lifespan. In addition to apoptosis and pruning, it is also worth noting that secondary mechanisms for neurological tissue removal also exist, such as necroptosis and pyroptosis, processes that are less common than apoptosis and are associated with potentially unhealthy tissue inflammation [30]. Thus, tissue removal in the human brain is expected to predominantly be the result of apoptosis and pruning. In this manuscript, it is theorized that both excessive and reduced tissue removal are potentially important factors in a variety of neurodevelopmental disorders, suggesting a bounded range of tissue removal that could constitute healthy brain development. Furthermore, sulcal formation and cortical folding occur early on in neurodevelopment; thus, any potential associated tissue removal-based role would have to occur during early development. However, it is known that tissue removal occurs throughout the lifespan, and so do additional aspects of MSCD, including gyrification and cortical thickness changes. Cortical thinning may reflect dendritic and glial changes, apoptotic processes, or a combination thereof. Thus, in this manuscript, we are considering the possibility that cortical thinning might be partially associated with tissue removal across the lifespan.

Existing theories of factors affecting MSCD include differential growth rates between gray matter (GM) and white matter (WM). This effect is extremely poignant early in life, with GM exhibiting decreases in older age. Skull constraints likely affect MSCD and clearly affect the brain across the lifespan. Axonal tension and pushing are expected to be important structural factors in the brain throughout the lifespan; however, their potential role in gyrification and sulcal formation may be quite pronounced but is also controversial.

This review article summarizes the main theories raised regarding mechanisms affecting MSCD, presents a theory connecting pruning and apoptosis to MSCD, relates findings from many studies on MSCD, including ones focused on several pathologies, and discusses the proposed theory’s potential relationship with pre-existing theories of MSCD.

## 2. Potential Factors in Macro-Structural Cortical Development

### 2.1. Spatially Varying Brain Growth

Differential growth rates of brain tissues have been identified as a potentially major contributing factor in cortical folding and sulcal formation [1,2,3,7,8,9], which are important largely during prenatal developmental milestones. The evidence in favor of differential growth rates contributing to the characteristic sulcal and gyral patterns of the cortex is extremely strong [2]. In this analysis, a physical model was constructed in which the simulated gray matter (GM) expands faster than the underlying simulated white matter (WM), replicating the differential growth rates of GM and WM, respectively, resulting in characteristic sulcal and gyral patterns forming in the outer cortical-like region [2]. Although their work, when repeated, produces similar patterns of fissures on the surface of their physical model, it is well known that much of the cortical folding and sulcal formation in the developing human brain must proceed in an extremely specific manner, as evidence suggests a relationship between sulcation and cognitive development [31]. As such, underlying mechanisms that direct sulcal formation, and potentially even cortical folding, might be needed in order to produce consistently healthy macro-cortical developmental patterns. It has also been suggested that differential proliferation of neural progenitors contributes to cortical folding and sulcal formation [7]. Additionally, the reader is also referred to a review of the empirical evidence suggesting that gyrification is the product of differential expansion of the cortex [8].

### 2.2. Skull Constraints

The constraining effects of the skull have been identified as a potential factor contributing to the macro-structural development of the human cortex [4]. Models of skull-related effects on cerebral cortical folding have been developed, and it has been reported that incorporation of skull constraints results in improved cortical folding simulation results [32]. Additional work on three-dimensional geometric modeling of brain development has suggested that mechanical constraints of the skull influence the cortical folding process [33]. Furthermore, advanced models based on finite element modeling and machine learning have demonstrated consistent predictions of cortical folding, implying that brain development can be predicted based on fetal brain configurations [34], with connected research implying the possibility for cranial constraints to potentially contribute to gyrification of the cortex [32,35]. It has been proposed that skull constraints and cerebrospinal fluid (CSF) pressure influence the developing brain and could contribute to cortical folding [36]. Additional research from developmental modeling has suggested that compressive constraints of the skull are not required for gyrification [37], whereas alternative models indicate it has an influential role [32,33]. Computational models are typically complex while attempting to model a system that is even more complex (“All models are wrong, but some are useful”—George Box), and so we cannot form firm conclusions about the potential role of the skull in sulcal formation and cortical folding based on models alone. However, by providing an inward-directed constraining force, the skull is expected to contribute to pressure and forces on the surrounding cerebrospinal fluid (CSF) and developing brain, which could potentially influence brain development. We also have evidence from craniosynostosis, a condition characterized by premature fusing of skull bones, causing growth restriction and associated intracranial anomalies detectable by magnetic resonance imaging (MRI) [38], implying a potential role for the skull in neurodevelopment.

### 2.3. Axonal Tension and Pushing

Theories have been developed on the potential role of axonal tension [5,6] and axonal pushing [10] in cortical folding and sulcal formation. It has been suggested that many structural features of the central nervous system can be explained by mechanical tension along axons, dendrites and glial processes [6]. The axonal tension theory suggests that axonal forces on linked neural tissue result in cortical convolutions [5]. Thus, it is theorized that “gyri form between strongly connected areas as axons draw them together, and sulci arise between weakly linked or unconnected areas” [5], and that by “keeping the aggregate length of axonal and dendritic wiring low, tension should contribute to the compactness of neural circuitry throughout the adult brain” [6]. Furthermore, it has been reported that “fibers pull together regions that are strongly connected, while unconnected regions drift apart” [5]. It has also been reported “that axonal fibers connected to gyri are significantly denser than those connected to sulci” [10], suggesting axonal pushing as a contributing factor in cortical folding [10]. While prior studies indicate the white matter could be an important factor in cortical folding [6], it has also been reported that a lack of cortical fibers induces folding due to buckling instability [3]. Thus, the axonal tension hypothesis implies that connections between cortical regions are under tension, causing buckling (see Figure 1 in [8] for visualizations).

Assessing the validity of the axonal tension theory is a major challenge, as tension measurements along an axon cannot be measured in vivo in the human brain as it develops, and the axonal tension theory’s role in cortical folding has been controversial [39]. As such, in assessing the theory, we often rely on computational modeling. Although a common opinion is that axonal tension is inadequate to cause gyrification, finite element modeling has indicated that axon tension can serve as a perturbation that triggers buckling in simulations, directing the location of resulting folding patterns [40]. Computational modeling does not, however, provide proof of the validity of the theory, and demonstrating causal mechanisms connecting axonal tension to cortical folding remains elusive.

### 2.4. Tissue Removal: Potential Role in MSCD

It is known that the human brain exhibits an excess of neurons at birth, estimated at 100 billion [41], leading to fewer in adulthood (estimates include 86 [42] and 67 [43] billion, reviewed in detail [44]). Apoptosis is the process of programmed cell death, in which a cell undergoes a controlled process of self-termination [45]. Thus, it is expected that across a healthy adult’s lifetime, the number of total neurons that underwent apoptosis should be measured in the billions. This manuscript is not the first to have suggested that apoptosis, or more specifically, rates of apoptosis, may have major effects on brain development. Indeed, accelerated apoptosis has been associated with microcephaly, an extremely serious condition associated with very low brain volumes, and decreased apoptosis has been associated with megalencephaly, a very serious condition associated with abnormally high brain volumes [18]. Thus, if apoptosis abnormalities are a major factor in extreme examples of high- and low-brain-volume neurodevelopmental conditions, it is plausible that healthy apoptosis, which proceeds at a range of rates across the population, potentially plays a significant role in MSCD. When apoptosis of a neuron occurs, connections between that neuron and surviving neurons are expected to be severed in the form of pruning. Pruning represents a critical component of brain maturation, is associated with maximizing memory performance, and has been reported to continue into at least the third decade of life [46]. Thus, the total volume of the neurons and all of their branches is expected to get smaller as pruning and apoptosis proceed. Thus, it is theorized that as these effects proceed over time, their cumulative effect is a contributing factor to MSCD. Additionally, it is hypothesized that abnormalities of tissue removal may also potentially play an important role in the development of pathologies beyond microcephaly and megalencephaly, potentially detectable at the macro-structural level. It is noteworthy that opioid use is associated with alterations to expression levels of apoptotic factors [47] in rat embryos and that opioids exposed pregnant women’s fetuses exhibit reduced gyrification [48], thus further emphasizing the potential for apoptotic processes to influence MSCD.

Since tissue removal is widespread across the lifetime, it is plausible that the MSCD of the brain would proceed quite differently had tissue removal not occurred and adults continued to have on the order of 100 billion neurons, as they did at birth. Thus, pre-existing theories, such as differential growth rates, skull constraints, and axonal tension, could represent an incomplete set of underlying factors contributing to MSCD.

#### 2.4.1. Tissue Removal: Potential Connection with Learning and MSCD

The brain is a circuit that exhibits electrical signal transmission [49], with a reduction in neurons later in life [42,43] relative to birth [41]. Thus, at birth, the brain closely resembles an over-wired circuit, with excess cells and associated connections, and closely resembles a more refined circuit by adulthood, with considerable tissue removal having occurred.

It is well known that healthy human brains undergo cortical thinning during development [16,50]. Pruning has been linked with memory function and learning [46,51]. Furthermore, macro-level cortical thickness changes observed with magnetic resonance imaging (MRI) have also been linked with memory function and learning [52,53,54], with both synaptogenesis and pruning potentially implicated in these processes. We observe increased rates of cortical thickness reductions during the developmental years [16] and reduced rates of cortical thickness reductions in adulthood [51]. Slower rates of learning are common in older individuals [55], and so known differential rates of cortical thickness reductions may be associated with learning rates in healthy individuals. Indeed, it has been reported in mouse models that the rate of pruning decreases with age [56]. Furthermore, previous research has reported that increases in intelligence (as assessed by the full-scale intelligence quotient—FSIQ) are associated with decreases in cortical thickness [57], providing further support for the potential existence of causal connections between learning, apoptosis and pruning, and cortical thinning. However, it should also be noted that cortical thinning, while being potentially associated with intelligence, is also associated with a variety of developmental conditions, some of which also exhibit intellectual disability, such as in multiple sclerosis [58,59,60], implying that cortical thinning in support of intelligence may be confined to a healthy range of developmental rates. Additional research on possible links between cortical thinning and intelligence is warranted.

It should be noted that while cortical thinning may be reflective of tissue removal, existing theories also implicate increased myelination of intracortical axons or changes in glial composition [61,62]; thus, tissue composition may play an important role in the thinning of the cortex. Ultimately, we may require a combination of factors, potentially including myelination and glial composition changes alongside tissue removal, in order to fully explain the thinning of the cortex.

The age-dependent rate of natural cortical thickness reductions has been observed to be correlated with the rate of increase in fractional anisotropy in the corresponding white matter (WM) of the frontal cortex, and it has been suggested that this phenomenon may be linked with pruning [63]. It is possible that the increased WM fractional anisotropy observed, and the decreased thickness observed in the cortex, are each associated with pruning in their respective tissues (i.e., removing extraneous WM axons can make the tissue more directed in its diffusion properties, potentially contributing to the observed fractional anisotropy changes). Similarly, lateral posterior cortical functional connectivity decreases were observed to be correlated with increases in white matter structural integrity of the corresponding cerebello-thalamo-cortical white matter tract, and it was suggested that this observed pattern could be linked with pruning [64].

Evidence from measuring protein expression levels in the cortices of mice that were and were not shocked to learn [65,66] is highly supportive of the theory that learning is linked with tissue removal [21]. This was a contextual fear conditioning experiment in which mice were placed in a chamber and then shocked so as to associate the shock with the chamber in which they were placed. Typically, mice, when placed again in the chamber, would freeze, demonstrating in outward behavior that they have learned to associate the chamber with the shock. The control mice were shocked before being placed in the chamber so that they would not learn to associate the chamber with the shock, yet they were still subjected to equivalent stress. After each experiment, the mice had a large series of protein expression levels measured from their cerebral cortices. Machine learning and feature selection technology were applied in a study completed in my lab [21], demonstrating that learning status (whether or not a mouse was shocked to learn) could be predicted by machine learning based on an identified subset of just six proteins, with 100% accuracy. Five of the identified proteins had previously been associated with learning: brain-derived neurotrophic factor (BDNF), the NR2A subunit of the N-methyl-D-aspartate receptor, histone H3 acetylation at lysine 18 (H3AcK18), protein kinase R-like endoplasmic reticulum kinase (pERK), and superoxide dismutase 1 (SOD1). The remaining protein identified, BCL2, is associated with apoptosis. Furthermore, BCL2, BDNF, and NR2A have all been associated with pruning. Thus, these findings are supportive of the theory that pruning and apoptosis are linked with learning.

The site of pruning and apoptosis is expected to significantly impact the effect on MSCD. Apoptosis of a single cell near the cortical surface, and associated pruning of connections directly associated with that removed cell, would be expected to have a small effect on the cortical surface that may accumulate with additional apoptotic events over time, potentially contributing to cortical thinning. However, if an upstream signaling pathway is removed, a substantial number of downstream neurons may lose their primary inputs, which could potentially lead to widespread localized apoptosis through their disuse due to the lack of inputs. Such an event could theoretically cause a broader regional reduction in neuronal packing density farther away from the surface of the brain, potentially playing a contributing role in sulcal formation and/or cortical folding. It is expected that any tissue removal contribution to cortical folding and major sulcal formation would be genetically modulated, with precise physiological developmental timing, in order for such phenomena to proceed in the same manner across the whole population, leading to nearly identical presentation of cortical folds and major sulci among healthy individuals. However, in the case of the development of minor sulci and cortical thinning, observed developmental patterns may be linked with experience-dependent tissue removal, producing large variation in cortical presentation across the healthy population.

Finally, it should also be noted that simulated apoptosis has also been demonstrated to contribute to regulation of learning and memory capabilities in adaptive artificial neural networks (ANNs) [67]. Additionally, adding adaptive neuron apoptosis to ANNs has been shown to improve training times and predictive accuracy in some applications [68]. Thus, findings from deep learning models also indicate that the software-modeled equivalent of tissue removal can be relied upon in advanced learning machines. Future research is warranted on the integration of the concepts of apoptosis and pruning in both deep learning models and computational neuroscience modeling of human brain development and learning.

##### A Potentially Illustrative Example from Electrical Engineering

The Field Programmable Gate Array (FPGA) [69] is an over-wired circuit widely used in digital hardware testing, allowing the user to specify arbitrary digital logic implemented on the FPGA by disabling key subcomponents of the over-wired circuit. The FPGA can implement any digital logic, which can implement any algorithm, including any learning algorithm at our disposal. Thus, an over-wired circuit with subcomponents disabled is clearly capable of implementing the fundamentals of learning. Thus, an over-wired brain that has tissue removed is theoretically capable of implementing learning, much like an FPGA. Learning proceeds throughout life (in some more than others), and the accumulation of tissue removal may have a significant impact on macro-structural cortical development (MSCD). The FPGA analogy outlined requires empirical validation and forms an interesting avenue for future research.

Patterns of spontaneous brain activity are associated with low rates of apoptosis in the cerebral cortex [70]. These findings imply that tissues that are metabolically active are much less likely to undergo apoptosis. Conversely, this also implies that tissues with low levels of activation are potentially more prone to neuron removal through apoptosis. Thus, these findings imply that apoptosis tends to occur in tissues not being substantially used/activated by the brain. Theoretically, an interconnected, over-wired circuit, responsible for learning, will initially have subcomponents of the circuit that contribute to erroneous predictions/undesirable outputs or behavior. Avoiding making errors in the future (thus demonstrably exhibiting the fundamentals of learning) could then be critically linked with not activating circuit subcomponents that have historically led to erroneous predictions/undesirable behavior. Although this discussion is speculative (and requires empirical validation), in the context of the human brain, this might imply that an individual practicing not making mistakes may eventually consistently not activate brain tissue that was leading to those mistakes. Eventually, that tissue might activate at such a low rate that it may be metabolically prioritized to undergo apoptosis and pruning. Thus, a potentially simple metabolic-based pruning and/or apoptosis factor has the potential to play an important role in learning by refining networks through disused tissue removal to be more metabolically efficient, while the individual practices not making mistakes. Removal of unused tissue may result in a functionally similar, more metabolically efficient network, a relatively simple property that could easily be genetically coded/directed.

#### 2.4.2. Tissue Removal: Potential Connection with Cerebrospinal Fluid

It has been proposed that skull constraints and cerebrospinal fluid (CSF) pressure influence the developing brain and could contribute to cortical folding [36]. The brain consists of a large quantity of soft tissue, with largely white matter fiber tracts throughout central regions of the brain connecting and relaying information between peripheral cortical gray matter, with a range of neuronal packing densities across brain regions and species [71]. The pial surface is the outer tissue at the external boundary of the cortical gray matter and is composed of the pia mater, arachnoid mater and dura mater. The pia mater is composed of fibrous tissue and acts as the meningeal envelope that firmly adheres to the surface of the brain. Research on tissue-level stresses and strains has reported that the range of physical properties for the pia mater has limited effects on the resultant stress and pressure in tissue [72], implying that the pia mater is pliable and deformable, and it is known to have elastic properties [73,74]. The pial surface separates the cortical gray matter from the cerebrospinal fluid (CSF), which exhibits a range of healthy pressures, with increased pressure being associated with neurological conditions [75], including its implication in the neurodevelopment of autism [76]. The CSF will necessarily exert an inward force on the brain. For every force there is an equal and opposite force; thus, the brain will necessarily exert an equal and opposite outward force towards the CSF. Indeed, models of neurodevelopment confirm brain expansion in the absence of a skull constraint [77], which is consistent with the brain exerting an outward force towards the CSF when it is in a stable state in vivo. When tissue is removed from the brain, through healthy processes of pruning and apoptosis, the reduction in tissue potentially results in a net reduction in neuronal packing density and internal connectivity, potentially resulting in a reduction in outward force exhibited by the brain towards the CSF, as there is less density of tissue remaining to exert a counterforce on the CSF. Thus, tissue removal is theorized to disrupt the equilibrium of forces along the brain’s surface. The resultant force imbalance is then theorized to contribute (potentially alongside slower tissue growth in the region subject to tissue removal) to the pial surface receding until settling into a new position that results in balanced inward and outward forces. The accumulation of many such examples of disruption to the equilibrium of forces along the brain’s surface is theorized to have a cumulative effect, potentially eventually contributing to changes at the macro-structural level, thus potentially playing a significant role in MSCD.

#### 2.4.3. Insights from Pathologies Potentially Characterized by Abnormally Reduced Tissue Removal

Megalencephaly is characterized by abnormally enlarged brains and is a very serious developmental disorder, for which researchers have suggested inhibited apoptosis as a potentially important factor contributing to the condition [18]. Mutation of genes linked with apoptosis has been implicated in the condition [20]. Hemimegalencephaly is a condition whereby megalencephaly presents on one of the two brain’s hemispheres, and analyses with magnetic resonance imaging (MRI) have demonstrated that non-affected hemispheres are smaller than in a control population [78], resulting in the hypothesis that each developing hemisphere is affected differently by mutations [78], potentially due to incomplete (or reduced) apoptosis [79].

The neurodevelopment of Down Syndrome has been studied at the macroscopic level, investigating cortical thicknesses in two studies [80,81], one of which was the product of my own research [81]. Findings demonstrate that young patients with Down Syndrome exhibit increased cortical thickness relative to healthy counterparts in both analyses [80,81]. This may imply delayed cortical thinning as a measurable brain phenotype potentially associated with intellectual disability in Down Syndrome and may be supported by the presented theory, implying that these observations are consistent with the theory of abnormal pruning being a characteristic of Down Syndrome (DS) patients. DS patients exhibit altered processing of amyloid precursor protein (APP) [82], potentially resulting in disrupted axonal pruning and neuronal culling [83]. DS patients have fewer microglia, an immune cell that is known to play an important role in synaptic pruning [84,85]. Additionally, dendritic branching, length, and spine density were reported to be increased in a young DS population [86,87], potentially implying a reduction in rates of pruning. Slower tissue removal due to slower pruning and/or apoptosis will result in less tissue removed, potentially impacting MSCD. Thus, pruning dysfunction in DS may play a contributing role in observed thicker cortices relative to neurotypical populations. MRI literature findings [80,81] indicate increased cortical thickness might be indicative of reduced/impaired pruning in DS, leading to the lessened age-dependent reductions in cortical thicknesses during childhood and adolescence reported. Reduced pruning may also give rise to abnormally reduced regional cortical thickness variability previously observed [81]. The pruning model presented implies that in patients with known pruning deficits, such as in Down Syndrome, we might observe reduced sulcal development as compared with a neurotypical population. Indeed, recent research has demonstrated reduced sulcal depth in living fetuses with Down Syndrome [88], providing support for the theory that reduced rates of tissue removal may contribute to reductions in sulcal development. Apoptosis has been considered as a potential developmental mechanism in Down Syndrome far less frequently than pruning. It should be noted that it has been reported that DS patients exhibit increased rates of apoptosis [89,90], including a mix of increases and decreases in apoptosis-linked proteins [89], potentially implying a dysregulation of natural apoptosis and pruning in the DS brain, possibly contributing to known intellectual disability in the condition.

MRI assessments of the brains of patients with attention deficit hyperactivity disorder (ADHD) have demonstrated abnormalities in both functional and structural connectivity that may arise from a common etiological mechanism involving pruning [91,92]. It has been reported that “There appears to be a 5- to 10-year lag in the pruning of fronto-striatal circuits in ADHD patients compared to their typically developing peers” [93], with reference to [94]. Reductions in normal pruning have previously been implicated as a possible abnormality associated with delayed development in ADHD [95]. However, it should be noted that there is some disagreement in the literature on the presentation of the cortex in ADHD from MRI [96,97,98], with findings reporting both increased and decreased cortical thicknesses. The condition is not as severe as many neurodevelopmental pathologies, and so we may observe smaller effect sizes than in other conditions, which may contribute to difficulties in reaching consensus on the phenotypic presentation of the ADHD brain, and may help explain disagreements in the observed MRI literature findings. Although the etiology of ADHD is clearly different from Down Syndrome, multiple studies have reported abnormally increased cortical thickness relative to healthy controls [99,100], as well as delayed peak thickness during puberty [101]. My own large-scale analysis of clinically imaged patients also reported increased cortical thickness in ADHD and confirmed the leading findings of cortical thickness increases in ADHD in a public domain dataset [102]. Analysis of patients from Boston Children’s Hospital indicates that larger thickness irregularities are observed in Down Syndrome [81] than those observed in ADHD [102], which might represent further support for the theory that pruning deficits are linked with intellectual disability, given that DS patients tend to exhibit more severe intellectual disabilities than those with ADHD. Thus, the ADHD literature potentially provides support for the theory that thicker cortices are observable with MRI in patients who exhibit reduced tissue removal. Within-subject regional reduced cortical thickness variability has also been observed in ADHD [103] relative to healthy controls, which might also be indicative of delayed pruning. Thus, it is theorized that we may see reduced sulcal development in key regions affecting attention and hyperactivity in ADHD, most likely in young age groups. Indeed, studies have reported on atypical sulcal patterns [104], as well as abnormal morphology of the central sulcus [105] in patients with ADHD, providing further support for the presented theory. It should also be noted that dopamine dysfunction has been potentially implicated in ADHD [103], and that dopamine has been previously linked to pruning [106], potentially providing further support for tissue removal abnormalities in ADHD. In the scientific literature, apoptosis has been considered in ADHD far less frequently than pruning; however, it has also been suggested that apoptosis may underlie selective brain region vulnerability in ADHD [107], potentially implying a role for apoptosis in affecting ADHD-based intellectual disability.

My colleague’s work investigating CHARGE syndrome (CS) has demonstrated cortical abnormalities [108]. As reported, “CHD7 is a responsive gene for CS” [108], and “heterozygous loss of CHD7 homologs affects gene expressions linked with axonal morphology such as axonal pruning” [108]. It was proposed that the observed increases in cortical thickness in CHARGE syndrome may be associated with pruning irregularities [108].

#### 2.4.4. Insights from Pathologies Potentially Characterized by Abnormally Increased Tissue Removal

Microcephaly is a very serious neurodevelopmental condition characterized by unusually small brains, and researchers have proposed that excessive apoptosis may be a major contributing factor in the etiology of the condition [18]. Additionally, gene mutations linked with apoptosis have been implicated in microcephaly [19]. A mouse model study has demonstrated that microcephaly can form due to increased developmental neuron death [109], further supporting the theory that the condition is linked with excessive tissue removal. In a broad general sense, tissue removal is expected to result in reduced outward force towards the CSF, which, when combined with largely unchanged inward forces exerted by the CSF, could potentially contribute to a smaller brain, a phenomenon that may be an important factor in MSCD in a variety of pathological conditions. In the context of healthy tissue removal, this could contribute to the thinning of the cortex. Thus, conditions with abnormally accelerated tissue removal may exhibit excessive cortical thinning.

Possible connections between abnormal tissue removal and schizophrenia have long been proposed, with specific focus on the possibility that schizophrenia may be characterized by pruning abnormalities in the prefrontal and temporal cortices [110,111,112,113], and may have an etiology that involves excessive pruning [111,112,113]. Macro-level analysis of the phenotypic presentation of the schizophrenic brain with MRI has demonstrated reductions in average cortical thicknesses [114,115,116,117,118,119,120,121], which includes two of my own analyses [120,121]. Thus, it is theorized that potentially accelerated tissue removal in schizophrenia could result in excessive cortical tissue loss and increased or irregular sulcal formation, potentially contributing to detectable decreases in average cortical thicknesses observed.

The literature has reported that increased diffusion heterogeneity was observed in the frontal lobes of patients with schizophrenia relative to healthy controls, and abnormal pruning was identified as a possible mechanism to explain these observations [122]. Given that diffusion is much less restricted in the direction of the length of an axon, when a branching fiber tract has some of its axons pruned, we should expect that region to often exhibit changes to the measured directed diffusion along the length of the pruned axons, which in turn could lead to the more heterogeneous spatial distribution of diffusion measurements observed [122]. It has also been observed that stressed mice exhibit hippocampal over-pruning [123], and schizophrenic patients have been reported to have abnormal structure and function of the hippocampus [124,125,126], including reduced hippocampal cortical thickness [127], which might be indicative of hippocampal-focused over-pruning. Based on the presented theory of pruning modulated MSCD, we might expect to observe abnormal sulcal and/or folding patterns in schizophrenia. Indeed, research has demonstrated that schizophrenia patients exhibit abnormal surface *inward* deformations in the bilateral anterior hippocampi [128], abnormal bilateral superior temporal, right inferior frontal and left calcarine sulci [129], and that the sulcal depth in the left hemisphere is correlated with the severity of symptoms related to working memory and executive function [130]. These findings are consistent with the theory of tissue removal being connected with observed abnormal deformations of the cortical surface, and potentially linked with critical aspects of learning, such as working memory. Literature results also indicated that genetic haploinsufficiency (a common occurrence in intellectual disability and schizophrenia) associated mutations caused a coordinated acceleration of pruning in neonatal cortical pyramidal neurons in a mouse model [131], implying possible genetic mechanisms associated with abnormal pruning, and could be associated with the abnormal phenotypic presentation of the brain in these conditions. Additionally, “increased pruning of primary neurites after exposure to the higher dose of dopamine” has been reported in schizophrenia patients [132]. Also of note, chronic cocaine use shares phenotypic similarities with schizophrenia, also exhibiting excessive cortical thinning, with thinner cortices associated with more severe symptoms [133]. As in the previously introduced conditions, the potential role of apoptosis in schizophrenia has not been considered nearly as thoroughly as the potential role of pruning; however, a dysregulation of apoptosis has been reported in key regions of the brain in schizophrenia [134], also potentially contributing to abnormal MSCD.

Findings have also implicated tissue removal abnormalities not only in schizophrenia but also in psychotic disorders more generally. Patients at ultra-high risk for psychotic disorders have been shown to exhibit distinctly lower cortical thicknesses of the insula [135], and the authors suggest that this might be linked with defective pruning. Thus, any potentially defective pruning identified [135] may specifically involve excessive pruning of axons projecting into the insula and/or excessive/irregular insular gray matter tissue apoptosis and pruning, potentially contributing to the thinner insular cortices observed.

Genetic haploinsufficiency (common in autism spectrum disorder) associated mutations caused a coordinated acceleration of pruning in a mouse model [131], indicating that there may be associations between autism spectrum disorder and tissue removal irregularities. Furthermore, cortical thickness irregularities have been observed extensively in autism [136,137,138,139,140]. My own research has also demonstrated irregularities of within-region cortical thickness variability in autism [139,140], findings that provide support for the possibility of tissue removal irregularities in autism that may be associated with increased rates of pruning and/or apoptosis, as well as potentially irregular distributions of sites of tissue removal. Indeed, over-pruning has previously been hypothesized as an abnormality characterizing autism [141,142], and an age dependency has been identified affecting axon pruning and myelination [29]; thus, macro-structural presentation of the brain via MRI may reflect this phenomenon. Additionally, literature studies have reported abnormal sulcal formation [143] and abnormal cortical folding [144] in autism. Apoptosis has also been implicated as being involved in the pathophysiology of autism [145,146], providing further support for abnormalities of tissue removal potentially playing an important role in MSCD in autism.

Microglial pruning has been reported to be abnormal in multiple sclerosis (MS) [147], potentially contributing to synaptic dysfunction. Additionally, the condition presents with abnormal structural development observed at the macro-level as imaged with MRI [148,149]. MS patients present with reduced cortical thicknesses [150,151], findings confirmed in my own analysis of the condition [152], and MS is known to exhibit demyelination of fiber tracts. In addition to abnormal microglial pruning, an MS-affected demyelinated neuron may, theoretically, provide reduced outward force as compared with a healthy neuron, as part of its structure, the myelin sheath, has become damaged or lost. Compromised structural integrity of a fiber tract could result in an abnormal reduction in outward force (towards the peripheral gray matter) from the entire branching tree structure that extends from that demyelinated fiber tract. Since white matter typically connects gray matter regions, this may result in reduced outward force broadly towards the gray matter. Apoptosis has also been implicated in multiple sclerosis [153,154], providing additional support for the potential for tissue removal to be linked with MSCD in MS. Potential reduced outward force due to microglial pruning, demyelination, and apoptosis, which, when combined with largely unchanged inward forces exerted by the CSF, could potentially contribute to a more compressed and thus thinner cortex, which is a well-known feature of MS [150,151,152]. It is noteworthy that an analysis has reported “age-associated cortical thinning correlated with transcriptomic profiles of genes involved in myelin and dendritic architecture” [15], potentially implying that major structural components of the brain, such as the myelin and the dendritic architecture, play a direct role in supporting healthy cortical thicknesses. Future work can investigate abnormal sulcal formation in MS. It is also noteworthy that recent research has suggested that there are differences in MS lesion formation between sites located in sulci and those located in gyri [155]; as such, further investigation of underlying abnormal neurodevelopment is warranted.

Cortical thinning associated with the severity of pediatric depression has been previously reported [156], and while this is not, in and of itself, evidence in favor of the presented theory on the role of tissue removal in MSCD, it is noteworthy that the authors suggest that experience-dependent pruning may be responsible for the observed effect [156].

Literature findings indicate that mouse model research demonstrates that genetically induced developmental axon pruning results in mice with decreased structural measurements of basal dendrites, who also exhibit epileptic seizures [157]. This research might imply that tissue removal may play a role in some seizure disorders (such as epilepsy), and literature findings have implied that pruning may be associated with a reduction in seizure generation [158]. Macro-structural phenotypic brain markers indicating abnormal levels of regional cortical thinning have also been observed in epilepsy, which might further imply that irregular pruning is associated with seizures [159,160]. It has also been reported that abnormalities of sulcal depth are observable in pediatric epilepsy [161], and apoptosis has also been linked with the condition [162], further supporting the theory that tissue removal may be linked with MSCD in a variety of pathological conditions.

A variety of additional conditions have also been implicated as potentially involving pruning and apoptosis abnormalities and may also present with macro-level irregularities in brain phenotype presentation. A diffusion-enabled MRI analysis of patients with idiopathic scoliosis (IS) demonstrates lower fractional anisotropy in the corpus callosum, findings that the authors indicate may be associated with abnormal pruning in the etiology of IS [163]. Fractional anisotropy is a measurement related to the degree to which the phase-shift-sensitive MR signal is spatially directed, and is calculated from a diffusion MRI exam. Tissue with highly directed profiles of the MR signal, such as white matter, exhibits considerable signal in the measurements that are oriented in parallel to the main fiber tracts in the white matter of the brain. Thus, reduced fractional anisotropy in the corpus callosum may be associated with excessive pruning and/or apoptosis.

#### 2.4.5. Tissue Removal: Potential Relationship with Pre-Existing Theories of MSCD

A variety of potential contributing factors to macro-structural cortical development have been outlined in this manuscript, in addition to the presentation of the theory of tissue removal’s potential role through pruning and apoptosis. Differential growth in the brain is highly likely to be an extremely important factor in MSCD. The gray matter is known to grow faster than the white matter, a process that naturally encourages folding on the cortical surface. While a model of brain development shows some consistency in folding patterns based purely on a physical model of simulated tissue growth [2], cortical folding patterns are well known to greatly affect functional development and intellectual abilities. A well-timed genetically regulated pruning and/or apoptotic process, occurring inwards of the position of a pending sulcus or fold, could potentially regulate regional development and help ensure healthy and consistent patterns of cortical folding and major sulcal development. Thus, pruning and/or apoptosis could play a role in guiding folding patterns and development of major sulci that would inevitably emerge due to the rapid growth of gray matter.

Although it is known that humans exhibit high consistency in the locations of major sulci and folds, there is high variability in the presentation of minor sulci across individuals. This could imply that genetically regulated mechanisms, potentially including pruning and/or apoptosis, underlie critical steps for developmental folding patterns and major sulcal development. Furthermore, minor sulci may form as a result of experience-based mechanisms, which may include pruning and apoptosis, and this may additionally be reflective of learning processes unfolding in the cortex. Axonal tension [5,6] and pushing [10] have also been identified as potential contributing factors to macro-structural development of the cortex. Indeed, based on the theory outlined, axons will inevitably exert forces on one another. Additionally, it has been reported “that axonal fibers connected to gyri are significantly denser than those connected to sulci” [10], which could imply that more substantial pruning and/or apoptosis has occurred in the axonal fibers connected to sulci relative to gyri in order to result in such a discrepancy. Thus, the fiber density differential between sulci and gyri [10] provides further support for tissue removal potentially being an important factor in sulcal formation.

Finally, skull constraints [4] have also been identified as a contributing factor in MSCD. Although the brain is typically surrounded by CSF separating it from the skull, the skull does play a critical role in maintaining the pressure of the CSF through its role as a mechanical constraint restricting outward expansion; thus, it certainly is expected to be a contributing factor in MSCD. Most likely, a complete model of MSCD will involve careful characterization of all of these potential contributing factors, along with their respective interactions with one another, in a unified model, a worthy future research avenue in computational modeling of brain development.

## 3. Conclusions

In summary, a theory has been presented implying a potential link between MSCD and tissue removal through apoptosis and pruning. Increased pruning factors are causally linked with reductions in the density and size of dendritic spines in mouse models [164]. A wide variety of conditions with known pruning and/or apoptosis irregularities exhibit abnormal macro-structural phenotypic presentation of the brain. Neuronal tissue removal is so widespread across an individual’s lifetime [41,42,43] that it could potentially have a major effect on MSCD. Future work will investigate the potential for a variety of pathologies with known pruning and apoptosis irregularities to be causally linked with MSCD abnormalities, such as aberrant folding, sulcal formation, gyrification, and cortical thinning.

## Data Availability

Not applicable.

## Abbreviations

The following abbreviations are used in this manuscript:

CSF	Cerebrospinal fluid
FPGA	Field programmable gate array
FSIQ	Full-scale intelligence quotient
GM	Gray matter
IS	Idiopathic scoliosis
MR	Magnetic resonance
MRI	Magnetic resonance imaging
MS	Multiple sclerosis
MSCD	Macro-structural cortical development
WM	White matter
